# Oncolytic Viro-Immunotherapy: An Emerging Option in the Treatment of Gliomas

**DOI:** 10.3389/fimmu.2021.721830

**Published:** 2021-10-05

**Authors:** Jiayi Zeng, Xiangxue Li, Max Sander, Haipeng Zhang, Guangmei Yan, Yuan Lin

**Affiliations:** ^1^ Zhongshan School of Medicine, Sun Yat-sen University, Guangzhou, China; ^2^ Peking University Sixth Hospital, Peking University Institute of Mental Health, NHC Key Laboratory of Mental Health (Peking University), National Clinical Research Center for Mental Disorders (Peking University Sixth Hospital), Peking University, Beijing, China; ^3^ Department of International Cooperation, Guangzhou Virotech Pharmaceutical Co., Ltd., Guangzhou, China; ^4^ Department of Pharmacology, School of Medicine, Jinan University, Guangzhou, China; ^5^ Department of Pharmacology, Zhongshan School of Medicine, Sun Yat-sen University, Guangzhou, China

**Keywords:** oncolytic virus, immunotherapy, delivery, combinations, gliomas

## Abstract

The prognosis of malignant gliomas remains poor, with median survival fewer than 20 months and a 5-year survival rate merely 5%. Their primary location in the central nervous system (CNS) and its immunosuppressive environment with little T cell infiltration has rendered cancer therapies mostly ineffective, and breakthrough therapies such as immune checkpoint inhibitors (ICIs) have shown limited benefit. However, tumor immunotherapy is developing rapidly and can help overcome these obstacles. But for now, malignant gliomas remain fatal with short survival and limited therapeutic options. Oncolytic virotherapy (OVT) is a unique antitumor immunotherapy wherein viruses selectively or preferentially kill tumor cells, replicate and spread through tumors while inducing antitumor immune responses. OVTs can also recondition the tumor microenvironment and improve the efficacy of other immunotherapies by escalating the infiltration of immune cells into tumors. Some OVTs can penetrate the blood-brain barrier (BBB) and possess tropism for the CNS, enabling intravenous delivery. Despite the therapeutic potential displayed by oncolytic viruses (OVs), optimizing OVT has proved challenging in clinical development, and marketing approvals for OVTs have been rare. In June 2021 however, as a genetically engineered OV based on herpes simplex virus-1 (G47Δ), teserpaturev got conditional and time-limited approval for the treatment of malignant gliomas in Japan. In this review, we summarize the current state of OVT, the synergistic effect of OVT in combination with other immunotherapies as well as the hurdles to successful clinical use. We also provide some suggestions to overcome the challenges in treating of gliomas.

## Introduction

Gliomas, which arise from glial or their precursor/stem cells, including diffuse gliomas and non-diffuse gliomas, are the most common primary CNS tumors (1, 2). Approximately 100,000 people around the world are diagnosed with diffuse gliomas every year (3). Based on WHO 2016 classification, diffuse gliomas can be further classified as: diffuse or anaplastic astrocytoma, isocitrate dehydrogenase (IDH)-wild type; diffuse or anaplastic astrocytoma, IDH-mutant; glioblastoma (GBM), IDH-wild type; glioblastoma, IDH-mutant; and oligodendroglioma or anaplastic oligodendroglioma, IDH-mutant and 1p19q co-deletion (4, 5). GBM, another term for WHO grade IV astrocytoma, is the most common type in adults and it is about four times more common than anaplastic astrocytoma (6). Currently, the treatment of gliomas faces great difficulties:

1. Gliomas have a poor prognosis after being treated with existing therapies (surgery, chemotherapy and radiotherapy) with median survival fewer than 20 months and a 5-year survival rate merely 4–5% (7, 8);
2. The presence of the BBB renders many conventional cancer drugs ineffective;
3. The immune-privileged environment in CNS makes immune checkpoint inhibitor therapy, which has been widely studied in recent years, less effective in gliomas (9);

Over the last decade, hopes have risen that emerging immunotherapy could improve specific immune responses against tumor cells in patients with brain tumors (10–13). Despite intensive clinical research, the FDA is yet to approve an immunotherapy for glioma.

Oncolytic Viruses can selectively or preferentially infect tumor cells and induce tumor lysis. Some of them, including parvovirus H-1 and reovirus, can also penetrate the BBB and possess tropism for the CNS, enabling intravenous delivery in clinical trials (14, 15). Furthermore, OVs can activate the innate immune response and the adaptive anti-tumor immunity to target distant uninfected tumors cells. As of 2021, at least 15 different virus species are currently under study: adenovirus, herpes simplex virus-1 (HSV-1), parvovirus, vaccinia virus, myxoma virus, reovirus, enterovirus, measles virus, Newcastle disease virus (NDV), vesicular stomatitis virus (VSV), retrovirus, Zika virus, M1 virus, Semliki Forest virus and Seneca Valley virus.

In this review, we will summarize the state of oncolytic virotherapy and its combination with other immunotherapies in gliomas.

## Immunosuppressive Microenvironment of Gliomas

The BBB consists of endothelial cells, astrocytes and pericytes, forming tight junctions to make the CNS an immune-privileged environment. In healthy individuals, most of peripheral immune cells are excluded from entering the brain (16). However, T cell entry and immunosurveillance within the brain have been documented over the years (17, 18).

**Figure 1:**
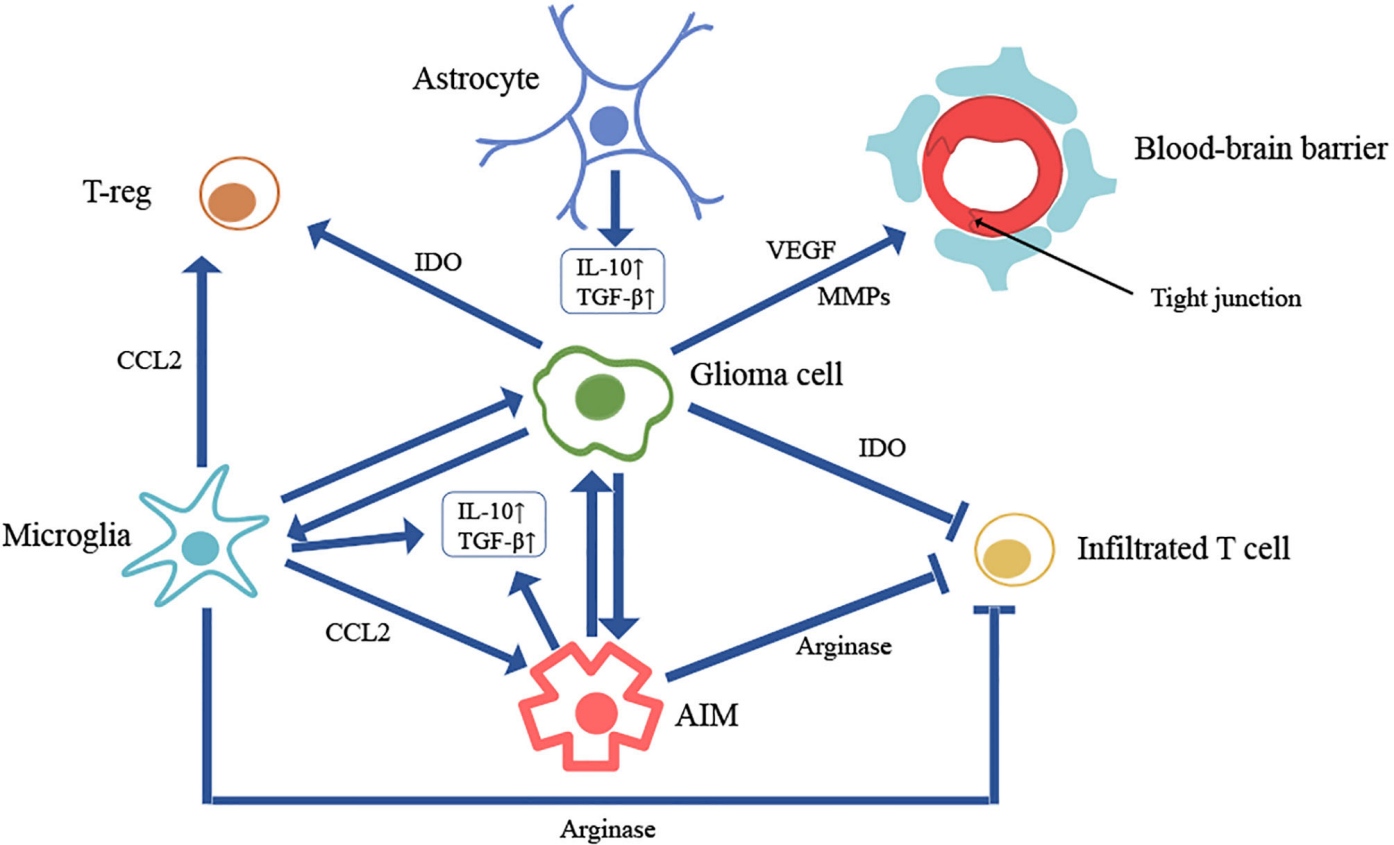
Cell interactions in brain tumor microenvironment. ***Microglia*** can 1) modulate glioma growth via facilitating proliferation, invasion and stemness of glioma; 2) promote the recruitment of T-reg cells and anti-inflammatory macrophages (AIM) from systemic circulation through the release of chemokine ligand 2 (CCL2); 3) produce high level of immunosuppressive cytokines like transforming growth factor β (TGFβ) and interleukin-10 (IL-10); 4) produce a large amount of arginase to inhibit T cell proliferation and function by depleting tissue arginine levels. ***AIM*** can 1) support tumorigenesis; 2) produce high level of immunosuppressive cytokines like TGFβ and IL-10; 3) produce a large amount of arginase to inhibit T cell proliferation and function by depleting tissue arginine levels. ***Glioma cell*** can 1) secrete soluble factors (VEGF, MMPs) to destroy the endothelial tight junctions, causing blood-brain barrier leakage; 2) generate a great amount of indolamine 2,3-dioxygenase (IDO) to both inhibit T cell activity and promote the recruitment of regulatory T cells (Treg) through depletion of tryptophan from the microenvironment; 3) attract both microglia and AIM to enhance tumor growth and promote immunosuppression. ***Astrocyte*** can also produce high level of immunosuppressive cytokines like TGFβ and IL-10.

During inflammation, particularly, microglia within the brain undergo substantial phenotypic changes, and specific macrophage populations are recruited from circulating monocytes (19). Microglia are CNS-resident myeloid cells that migrate to the developing brain early in gestation (20). They are responsible for phagocytosis and synapse formation and pruning in both healthy and pathological states (21, 22). More and more preclinical and clinical studies found that microglia can modulate glioma growth *via* facilitating proliferation, invasion and stemness of gliomas (23–26). Furthermore, microglia can promote the recruitment of T-reg cells and anti-inflammatory macrophages from systemic circulation through the release of chemokine ligand 2 (CCL2) (27). Anti-inflammatory macrophages (AIM) in gliomas have reciprocal effects with microglia on enhancing tumorigenesis (28). AIM in gliomas are also suggested to support angiogenesis and mediate glioma recurrence (29, 30). Therefore, glioma-associated microglia and macrophages can be developed as therapeutic targets for glioma patients (31) (**Figure 1**).

It is also well-documented that tumors can secrete soluble factors in high concentrations, such as vascular endothelial growth factor (VEGF) and matrix metalloproteinases (MMPs). These factors can destroy the endothelial tight junctions, degrade proteoglycans and induce numerous blood derived factors (32, 33). A significant challenge in treating brain tumors is to enable a drug to cross the BBB so the breakdown of the endothelial tight junctions can be an opportunity to deliver drugs to gliomas.

Despite increasing evidence showing that the notion of “immune-privileged” is inaccurate, brain tumors still can prevent the immunosurveillance of the CNS and foster antigenic ignorance - for example, in sphingosine 1 phosphate receptor 1 (S1PR1)-dependent fashion. S1PR1 is a G protein-coupled receptor (GPCR) that binds sphingosine-1-phosphate (S1P), a lipid second messenger. S1P-S1PR1 axis plays an important role in lymphocyte trafficking. The level of S1P1 on the surface of T-cells in GBM patients is decreased compared to healthy controls. The dysfunction of this axis results in T-cell trapping within lymphoid organs, preventing T-cells from trafficking to the brain (34, 35).

Owing to the vulnerability of the brain to changes in intracranial pressure, an immunosuppressive environment inhibits the development of intracranial inflammation, including tumor-related inflammation (36). Various cells (AIMs, microglia, astrocytes) in tumor microenvironment can produce high level of immunosuppressive cytokines like transforming growth factor β (TGFβ) and interleukin-10 (IL-10) in response to inflammatory stimuli (18, 37, 38). Glioma cells can generate a great amount of indolamine 2,3-dioxygenase (IDO) to both inhibit T cell activity and promote the recruitment of regulatory T cells (Treg) through depletion of tryptophan from the microenvironment (39). Also, glioma cells attract both microglia and AIM to enhance tumor growth and promote immunosuppression (23). A large amount of arginase can be produced by microglia and tumor-infiltrating myeloid cells to inhibit T cell proliferation and function by depleting tissue arginine levels (18, 40, 41). Relatively low mutation load, little T cell infiltration and immunosuppressive microenvironment have been observed in GBM, making for a “cold” tumor microenvironment (10). It is generally accepted that this “cold” microenvironment makes ICIs less effective in the treatment of GBM (42).

## Oncolytic Virotherapy for Gliomas

### Oncolytic Virus

**Figure 2:**
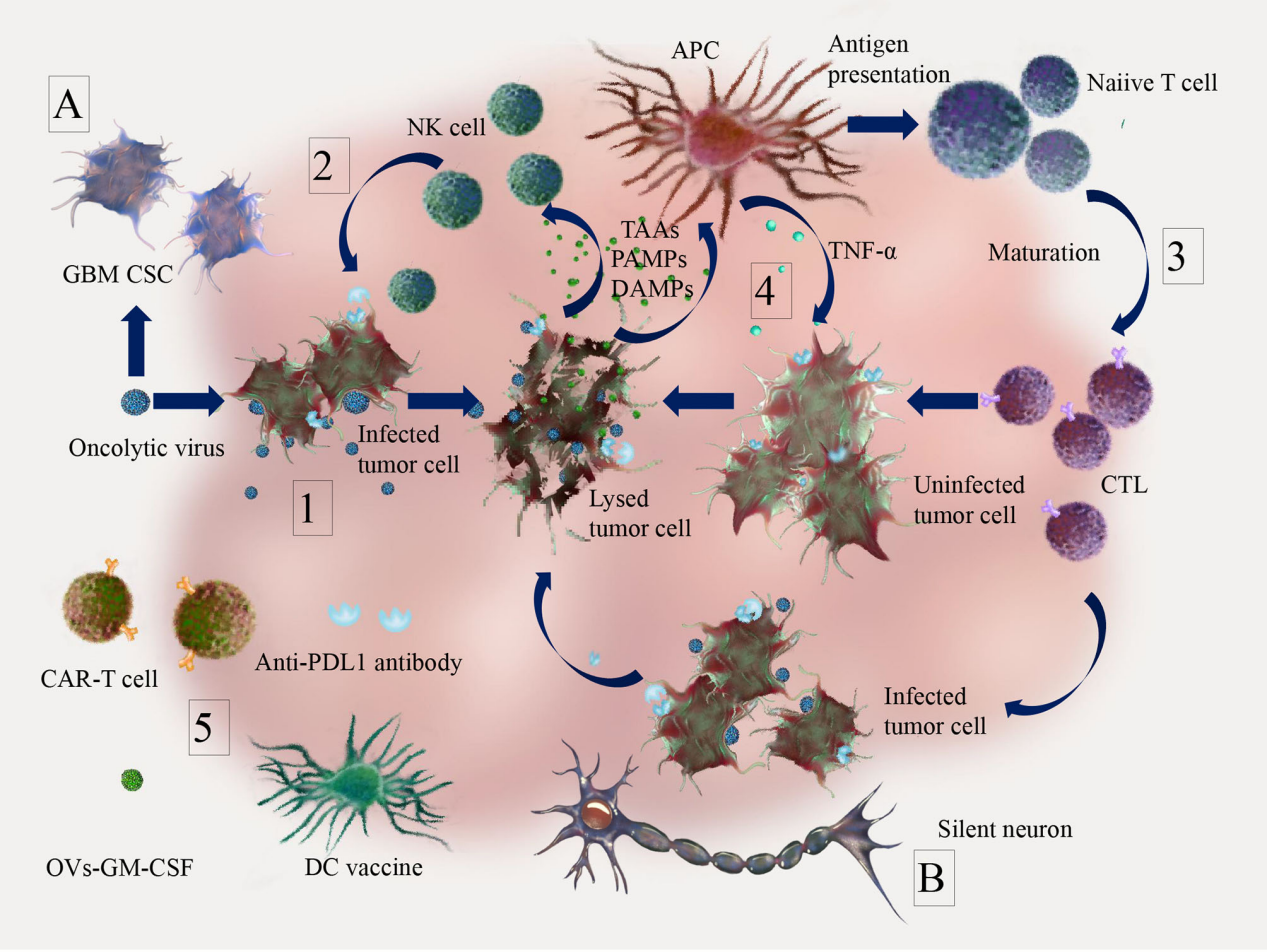
Anti-tumor effects of oncolytic virus (OV) and combination therapy in brain tumor. 1. OVs can selectively or preferentially infect tumor cells and induce tumor lysis. 2. Innate immune response. Tumor cell lysis due to OVs infection can cause the release of tumor associated antigens (TAAs), cell-derived damage-associated molecular patterns (DAMPs) and viral pathogen-associated molecular patterns (PAMPs), which can recruit dendritic cells (DCs) and innate lymphoid cells (e.g. NK cells) for early clearance of virus-infected cells; 3. Adaptive immune response. The release of TAAs, DAMPs, PAMPs, pro-inflammatory cytokines and chemokines by lysed tumor cells can trigger activation of antigen presenting cells (APCs) and promote the priming of cellular mediated immune responses (CTL infiltration); 4. OVs infection leads to the release of TAAs, PAMPs and DAMPs, which can induce innate immune responses (e.g. secretion of TNF-α) against not only infected tumor cells, but also uninfected tumor cells through bystander effects; 5. Infection and replication of oncolytic viruses in tumors can activate anti-tumor immunity and turn “cold” into “hot” tumors, which make combination therapies such as immune checkpoint inhibitors (e.g. PD-1/PD-L1 inhibitor), adoptive cell therapy (e.g. CAR-T), tumor vaccines (e.g. DC vaccine) and immunotherapeutic modulators (e.g. GM-CSF, which can enhance the activation of NK cells and CD8-mediated T cell response) more effective. For glioma specifics, **(A)** antiviral innate immunity pathways (IFN pathway, TLR pathway) are reduced in glioblastoma cancer stem cells (GBM CSC), which contributes to the tumor cell specificity of OVs. **(B)** Due to the isolated location surrounded by mitotically silent normal neurons, malignant gliomas may be particularly suitable for treatment with OVs, which require active cell cycles for their replication.

Oncolytic viruses are an emerging class of antitumor immunotherapies. Talimogene laherparepvec (T-Vec), a genetically engineered OV based on HSV-1, is the most prominent and the only FDA approved OV used for treating malignant melanoma (43). OVs can selectively or preferentially infect and kill tumor cells, while activating the immune system (44) (**Figure 2**), by the following general mechanisms:

1. OVs can selectively or preferentially infect tumor cells and induce direct tumor lysis due to the deficient or inhibited antiviral innate immunity pathways (e.g. IFN pathway) in many tumor cells (45, 46).
2. Tumor cell lysis due to OV infection can cause the release of tumor associated antigens (TAAs), cell-derived damage-associated molecular patterns (DAMPs) and viral pathogen-associated molecular patterns (PAMPs), which can recruit dendritic cells (DCs) and innate lymphoid cells (e.g. NK cells) for early clearance of virus-infected cells (47).
3. The release of TAAs, DAMPs, PAMPs, pro-inflammatory cytokines and chemokines by lysed tumor cells and innate immune cells can promote antigen presentation and antigen-specific adaptive immune responses (48, 49).
4. The immune responses kill not only infected tumor cells, but also uninfected tumor cells through bystander effects (50, 51).
5. OVs can promote the recruitment of tumor infiltrating lymphocytes into tumor sites, making the immunosuppressive microenvironment “hot” and suitable for other immunotherapies (52).

In the following, the particularities of these mechanisms in glioma are discussed. In glioblastoma cancer stem cells, antiviral innate immunity pathways (IFN pathway, TLR pathway) are reduced, which contributes to the tumor cell specificity of OVs (mechanism 1) (53, 54). Interestingly, due to the isolated location surrounded by mitotically silent normal neurons, malignant gliomas may be particularly suitable for treatment with OVs, which require active cell cycles for their replication (55, 56). Utilizing mechanisms 2-5, a growing body of evidence suggests that OVs can “heat” the immunologically “cold” micro-environment of GBM by inducing immunogenic cell death (ICD) and inflammation. In a large number of preclinical studies, ICD was induced by OVs, along with infiltration of cytotoxic T cells and reduced accumulation of myeloid-derived suppressor cells (MDSCs) (57, 58). Clinical studies have also shown that OVs can induce cytotoxic T cells infiltration and other relevant immune responses against glioma (59–61). Further research is needed to understand mechanisms of interaction of components in the tumor microenvironment to fully exploit the potential of OVs in glioma. Modern discoveries related to the molecular mechanisms of specific OVs are discussed in paragraphs “*DNA Viruses*” and “*RNA Viruses*”.

When considering OVs for glioma treatment, it is vital to exclude OVs which display neurotoxicity. With few exceptions, these viruses fall into one of two groups: 1) non-neurotoxic viruses and 2) neuro-attenuated viruses. Non-neurotoxic viruses are viruses that have not been observed to be neurotoxic and do not necessarily require additional engineering before being used for treatment, including parvovirus, myxoma virus, M1 virus and Seneca Valley virus (SVV) (62). Neuro-attenuated viruses are viruses with neurovirulent features that have been modified (e.g. by gene editing) to reduce neurotoxicity. Adenovirus, herpes simplex virus (HSV), VSV, poliovirus and measles virus fall under this category. The various mechanisms of viral attenuation leading to decreased neurotoxicity are discussed below.

In the case of HSV, a number of mutants were created by modifying the virus to reduce neurovirulence without affecting the virus’s ability to infect actively dividing cells (63). For example, HSV-1716 and R3616 mutants have deletions in both copies of the viral γ_1_34.5 genes, which are necessary for neurovirulence (64, 65). The neurovirulence of poliovirus is attributed to the following two aspects: 1) selective binding of CD155 receptor-expressing motor neurons and 2) an internal ribosomal entry site (IRES) sequence in the viral genome. The neurotoxic poliovirus was engineered into a neuro-attenuated virus by replacing the entire IRES with its non-pathogenic counterpart from human rhinovirus type 2 (66). Several mutants have been created to reduce the potential neurotoxicity of VSV in the treatment of glioma, studied in a rodent model (67, 68).

In this review, we will discuss 15 different virus species which are currently under study to treat gliomas. Among them, 5 are DNA viruses, while 10 are RNA viruses. Important features of these viruses have been listed in **Table 1**.

**Table 1:** Important features about the oncolytic viruses mentioned above.

Virus type	Family	Genome	Genome size	Transgene capacity	Viral immunogenicity	BBB penetration	Ref
Adenovirus	Adenoviridae	dsDNA	32kb	High	Low	–	(69, 70)
Herpes simplex virus	Herpesviridae	dsDNA	152kb	High	Low	–	(70, 71)
Parvovirus	Parvoviridae	ssDNA	5kb	Low	High	+	(70, 72)
Vaccinia virus	Poxviridae	dsDNA	190kb	High	High	–	(73, 74)
Myxoma virus	Poxviridae	dsDNA	161.8 kb	High	High	–	(74, 75)
Reovirus	Reoviridae	dsRNA	23kb	Low	Low	+	(70, 76)
Enterovirus	Picornaviridae	ss(+)RNA	7.2-8.4kb	Low	Moderate	+	(70, 77)
Measles virus	Paramyxoviridae	ss (–)RNA	16kb	Low	Moderate	–	(78, 79)
Newcastle disease virus	Paramyxoviridae	ss (–)RNA	15kb	Low	Low	+	(79, 80)
Vesicular stomatitis virus	Rhabdoviridae	ss (–)RNA	11kb	Low	Low	–	(79, 81)
Retrovirus	Retroviridae	ss(+)RNA	7–10 kb	Moderate	Low	+	(79, 82)
Zika virus	Flaviviridae	ss(+)RNA	10.7kb	Low	High	+	(79, 83, 84)
M1 virus	Togaviridae	ss(+)RNA	11.7kb	Moderate	Moderate	+	(79, 85)
Semliki Forest virus	Togaviridae	ss(+)RNA	13kb	Moderate	Moderate	+	(79, 86)
Seneca Valley virus	Picornaviridae	ss(+)RNA	7kb	Low	High	+	(70, 87)

dsDNA, double-stranded DNA; ssDNA, single-stranded DNA; dsRNA, double-stranded RNA; ss(+)RNA, positive single-stranded RNA; ss(-)RNA, negative single-stranded RNA; Transgene capacity, the maximum size of inserted foreign gene fragments, Low(<7kb), Moderate(7-10kb), High(>10kb); Viral immunogenicity, the strength of immune response to the oncolytic virus backbone and the transgene(the Low-Moderate-High comparison is based on the capacity of virus induced antibodies); BBB penetration, (+) with study validation, (-) without study validation.

### DNA Viruses

We have listed the current clinical trials utilizing DNA viruses against gliomas in **Table 2**.

**Table 2:** Current clinical trials utilizing DNA viruses against gliomas.

Virus type	Strain	Targeted malignancy	Routes	Latest phase	Combination therapy	Trial No.	Status
Adenovirus	SCH-58500	Brain tumor	IT	I	Conventional surgery	NCT00004080	Completed
	DNX-2440	Glioblastoma	IT	I		NCT03714334	Recruiting
	CRad-S-pk7	Brain tumor	IC	I	Neural stem cells loaded with an oncolytic adenovirus	NCT03072134	Active, not recruiting
	DNX-2401	Glioblastoma/Recurrent Tumor	IT	I	Temozolomide	NCT01956734	Completed
		Glioblastoma or Gliosarcoma	IT	I	IFN-γ	NCT02197169	Completed
		Recurring Glioblastoma	IT	I		NCT00805376	Completed
		Recurring Glioblastoma	IT(CED)	I/II		NCT01582516	Completed
		Brain tumor	IT	II	Pembrolizumab	NCT02798406	Active, not recruiting
		Recurrent Glioblastoma	IA	I		NCT03896568	Recruiting
HSV	C134	Brain tumor	IT	I		NCT03657576	Recruiting
	M032	Brain tumor	IT	I		NCT02062827	Recruiting
	rQNestin 34.5	Brain tumor	IT	I		NCT03152318	Recruiting
	G207	Brain tumor	IT	I	Radiation	NCT02457845	Active, not recruiting
		Brain tumor	IT	I/II		NCT00028158	Completed
		Pediatric brain tumor	IT	I	Radiation	NCT03911388	Recruiting
		Pediatric brain tumor	IT	II	Radiation	NCT04482933	Not yet recruiting
	HSV-1716	Pediatric brain tumor	IT	I	dexamethasone	NCT02031965	Terminated
	G47Δ	Residual or recurrent glioblastoma	IT	II		UMIN000015995	Completed
Vaccinia virus	TG6002	Glioblastoma	IV	I/II	5-flucytosine	NCT03294486	Recruiting
Parvovirus	H-1PV	Glioblastoma	IV, IT, IC	I/II		NCT01301430	Completed

IT, Intratumoral; IC, Intracavitary; IV, Intravenous; IA, Intra-arterial; CED, Convection-enhanced delivery. (The data is based on “clinicaltrials.gov”).

#### Adenovirus

Adenoviruses are double-stranded-DNA viruses of the *Adenoviridae* family with 70–90 nm in size (88). The adenovirus has sufficient transgene capacity which can carry therapeutic genes of sizes of about 30–38 kb (69). A genetically modified variant, AdDelta24-RGD, also known as DNX-2401, has the ability to selectively infect glioma cells after either intratumoral or intracavitary (injection into the surgically created resection cavity) delivery (59).

Safety in patients with recurrent high-grade glioma has been demonstrated in phase I clinical trial (NCT00805376). In this study, an improved median overall survival has been shown in patients who received surgical resection as well as AdDelta24-RGD. Seven patients had a long-term survival of over 24 months and no Grade 3 or greater adverse events occurred (59, 89). Another phase I trial demonstrated safety in patients treated with AdDelta24-RGD through convection enhanced delivery (CED), an intratumoral delivery using continuous, low–positive-pressure bulk flow to deliver drugs through the implantation of catheters (90–92). AdDelta24-RGD is currently studied as a combination therapy in multiple phase I and II studies (NCT01956734, NCT02197169, NCT02798406).

CRAd-S-pk7 is an oncolytic adenoviral vector containing a survivin promoter and a pk7 fiber modification to selectively target glioma (93). A recent study found that it could be successfully encapsulated within mesenchymal stem cells (MSCs) as a new delivery strategy to treat diffuse intrinsic pontine glioma (DIPG), one of the deadliest brain tumors in children (94). Another phase I clinical trial using neural stem cells (NSCs) as delivery vehicles is ongoing (NCT03072134).

High levels of chronic immune activation detected in cancer patients correlate with poor prognosis in treatment with oncolytic adenovirus. Therefore, it might be necessary to screen the immune status of patients before treatment (95, 96).

#### Herpes Simplex Virus

Herpes simplex virus-1 is a double-stranded, linear-DNA virus of the *Herpesviridae* family that has been widely adopted for OVT and most extensively studied (71). An advantage of HSV-1 is that it can incorporate multiple large transgenes within its genome. In preclinical studies, by incorporating transgenes encoding immunomodulatory molecules, such as interleukin 12 (G47Δ-mIL12), oncolytic HSV-1 has been shown to greatly enhance the efficacy of treatment in a glioblastoma model (97, 98). An HSV-1 based intralesional oncolytic immunotherapy, T-Vec, has been approved by EMA and FDA to treat unresectable melanoma in adults (99).

G207 and HSV-1716 are two HSV-1 variants with the ability to target and kill glioma cells. Phase I and II trials have investigated their safety as monotherapies and combined with radiotherapy, with no serious adverse events documented (100–102). In particular, a phase I clinical trial using G207 alone and with radiation to treat pediatric high-grade gliomas recently reported no dose-limiting toxicity or serious adverse events (103). In addition, an interleukin-12 expressing HSV-1, M032, is being evaluated for the safety and tolerability of the maximum dose in patients with recurrent gliomas in a phase I clinical trial (NCT02062827).

G47Δ is another oncolytic HSV-1 strain developed by introducing another deletion mutation to the genome of G207 (104). G47Δ has a strong induction of antitumor immunity, and it has been shown to kill cancer stem cells derived from human glioblastoma efficiently (105). G47Δ is greatly attenuated and therefore expected to be safer than G207 and T-Vec in normal tissues (105). Recently, a single-arm phase II clinical trial in Japan was completed to test the efficacy of G47Δ administered stereotactically in patients with residual or recurrent glioblastoma. Side effects were limited and the 1-year-survival rate of 13 patients has reached 92.3% (UMIN000015995) (106). Based on this phase II trial, G47Δ (Delytact/Teserpaturev) has received conditional approval from Japan’s Ministry of Health, Labour and Welfare (MHLW) as an oncolytic virotherapy for the treatment of patients with malignant glioma in Japan. This certainly represents a breakthrough for OVT in glioma and the publication of the detailed results of the Phase II trial that led to approval are eagerly awaited by the scientific community.

#### Parvovirus

Parvoviruses are small, single-stranded DNA viruses of the *Parvoviridae* family (72). One variant, H-1, is the smallest among all OVs and it has potential for intratumoral and intravenous application. It is suitable for oncolytic virotherapy of brain tumors due to its capacity to cross the BBB (14, 107, 108). It has been shown to be effective in rat and human GBM cell lines (14). Besides, it has been shown to increase DC cross-presentation of tumor antigens in a melanoma cell line, demonstrating its ability to boost host immune reactivity (109). In glioblastoma patients, H-1 treatment was safe and triggered immunogenic changes in the tumor microenvironment in a phase I/II clinical trial (NCT01301430) (110).

#### Vaccinia Virus

Vaccinia viruses are large, enveloped, double-stranded-DNA viruses of the *Poxviridae* family. Strong transgene capacity (up to 40kb), efficient life cycle and selective replication in cancer cells make vaccinia viruses very promising for use as an OVT (73). Double deleted vaccinia virus (vvDD), with deletions of the thymidine kinase and vaccinia growth factor genes to enhance the safety profile, could preferentially infect and kill both Temozolomide (TMZ) resistant human brain tumor stem cells (BTSCs) and differentiated compartments of GBMs *in vitro*. Therefore, vvDD can be used as an effective supplement in the treatment of glioma, particularly for GBM patients resistant to TMZ (111, 112).

In a recent study, vvDD, expressing the fusion protein IL15Rα-IL15 and a fluorescent protein, was used to treat murine glioma GL261 *in vitro* and *in vivo* in combination with other treatments, including chemotherapy, peptide vaccine and adoptive T cell therapy (ACT). Pre-clinical results show potent antitumor effects against brain tumors when combined with celecoxib, rapamycin and ACT (113). To date, there are no clinical trials of VV in patients with glioma.

#### Myxoma Virus

Myxoma virus (MYXV) is also a double-stranded-DNA virus of the *Poxviridae* family (75). MYXV infects only rabbits in nature and is non-pathogenic to humans. More importantly, MYXV can preferentially infect and kill cancer cells originating from humans (75). MYXV has shown potent oncolytic activity in experimental human gliomas and produced a synergistic effect when combined with rapamycin in an immunocompetent glioma model (114, 115). It can promote natural killer (NK) cell mediated lysis of malignant gliomas both *in vitro* and *in vivo* (116).

MYXV could infect and kill both TMZ-resistant and -sensitive brain tumor-initiating cells (BTICs), which retained stem-cell-like properties (117). Anti-apoptotic M011L-deficient MYXV induced apoptosis in BTICs and prolonged animal survival in an immunocompetent glioblastoma model (118). Multiple compounds, e.g. axitinib, that synergize with oncolytic MYXV against human BTICs were identified (119). In immunocompetent animal models of glioma, MYXV armed with IL15Rα-247 has been shown to be a safe and powerful agent against brain tumors when combined with other immunotherapeutic methods (120). To date, there are no clinical trials of MYXV in patients with glioma.

### RNA Viruses

We have listed the current clinical trials utilizing RNA viruses against gliomas in **Table 3**.

**Table 3:** Current clinical trials utilizing RNA viruses against gliomas.

Virus type	Strain	Targeted malignancy	Routes	Latest phase	Combination therapy	Trial No.	Status	
Measles Virus	MV-CEA	Brain tumor	IT/IC	I		NCT00390299	Completed
Poliovirus	PVSRIPO	Pediatric brain tumor	IT(CED)	I		NCT03043391	Recruiting
		Malignant glioma	IT (CED)	I		NCT01491893	Active, not recruiting
		Malignant glioma	IT (CED)	II		NCT02986178	Active, not recruiting
		Recurrent glioblastoma	IT (CED)	II	pembrolizumab	NCT04479241	Recruiting
Reovirus	REOLYSIN	Pediatric brain tumor	IV	I	Sargramostim	NCT02444546	Active, not recruiting
	REOLYSIN	Malignant glioma	IT	I		NCT00528684	Completed
Retrovirus	Toca 511	Recurrent high-grade glioma	IC	II/III	5-fluorocytosine	NCT02414165	Terminated
	Toca 511	Recurrent high-grade glioma	IC	I	5-fluorocytosine	NCT01470794	Completed
	Toca 511	Recurrent high-grade glioma	IC	I	5-fluorocytosine	NCT01985256	Completed
	Toca 511	Recurrent high-grade glioma	IT/IV	I	5-fluorocytosine	NCT01156584	Completed

IT, Intratumoral; IC, Intracavitary; IV, Intravenous; CED, Convection-enhanced delivery. (The data is based on “clinicaltrials.gov”).

#### Reovirus

Reovirus is a double-stranded RNA virus of the *Reoviridae* family and is 75–85 nm in size with an icosahedral capsid (76). Reolysin is a reovirus-based agent which has shown the ability to penetrate the BBB, with specificity and oncolytic activity for glioma cells *in vitro* and *in vivo* (15, 121). In a preclinical glioma model, reovirus can up-regulate IFN-regulated gene expression and activate PD-1/PD-L1 axis in tumors suggesting that the reovirus combined with ICIs may enhance systemic therapy (15).

In a phase I dose escalation trial, dose limiting toxicities were not identified and a maximum tolerated dose was not reached (122, 123). A current phase I study of intravenously injected reovirus combined with granulocyte macrophage colony-stimulating factor (GM-CSF) is ongoing in pediatric patients with high-grade relapsed or refractory brain tumors (NCT02444546). Reolysin (Pelareoreop) has been granted orphan drug status by US FDA for the treatment of brain cancer (124).

#### Enterovirus

Poliovirus (PV) is a single-stranded, positive-sense RNA enterovirus of the *Picornaviridae* family and the cause of poliomyelitis (77). An attenuated poliovirus variant, PVS-RIPO, remains oncolytic but does not cause poliomyelitis and displays no neurovirulence in nonhuman primates (66, 125). It has been shown in animal models that a strong inflammatory reaction against infected glioma cells was triggered after PVS-RIPO inoculation (126, 127). In another preclinical study, PVS-RIPO improved OS in glioma-bearing mice (128).

Enterovirus A71 (EV-A71) has recently been found to have the potential to treat malignant gliomas. EV-A71 can selectively infect and kill malignant glioma cells, thus inhibiting tumor growth in mice (129).

In clinical studies, intratumoral injection of PVS-RIPO in patients with recurrent WHO grade IV malignant glioma was determined to be safe and improved OS (130). Currently, a phase Ib pediatric clinical trial is investigating the safety and dosage of PVS-RIPO by CED in high-grade gliomas as well as other brain tumors (NCT03043391). Ongoing phase II trials are also evaluating PVS-RIPO as a monotherapy or in combination therapy with pembrolizumab (NCT02986178) (NCT04479241).

#### Measles Virus

Measles virus (MV) is a single-stranded, negative-sense RNA virus of the *Paramyxoviridae* family. Unlike the wild type MV strains, attenuated vaccine strains have adapted to bind CD46 receptor for target cell entry (78). Normal brain tissue expresses low-level CD46, but glioma cells express abundant CD46 receptors on their surface. CD46 mediates MV attachment, internalization and virus-induced cell-to-cell fusion, and the safety of MV has been established in nonhuman primates (131, 132).

MV-CEA is a modified MV expressing carcinoembryonic antigen (CEA). CEA can serve as a marker of viral gene expression as the virus replicates. A pre-clinical study found that MV-CEA could induce brain tumor regression and improve OS in mice (133). MV-CEA could up-regulate PD-L1 in human GBM cells, and the combination with PD-1 blockade showed an increase in CD8+ TILs and improved OS in mice (134), supporting the potential of clinical combination of MV with αPD-1 therapy in GBM treatment (135). A phase I clinical trial of MV-CEA for the treatment of recurrent glioblastoma multiforme found no dose limiting toxicities with MV-CEA doses up to 2×10^7^ TCID50 (NCT00390299) (136).

Oncolytic MV encoding thyroidal sodium iodide symporter (MV-NIS) can enable *in vivo* tracking of MV infection and enhance therapeutic efficacy (137). Cells infected by MV-NIS can express NIS. which allows cells to actively transport iodide ions into the cells, providing a possibility for *in vivo* radioiodine imaging studies (138). It is possible to use NIS as a non-immunogenic marker for viral gene expression in the future. A phase I clinical trial evaluating the safety and recommended phase 2 dose of MV-NIS for the treatment of recurrent medulloblastoma or atypical teratoid rhabdoid tumor (ATRT) in children and young adults is recruiting (NCT02962167).

#### Newcastle Disease Virus

NDV is a large, single-stranded, negative-sense RNA virus of the *Paramyxoviridae* family (80). It has the ability to induce apoptosis of host cells *via* mitochondrial pathway and activate the antitumor immune response *via* increasing TNF-α secretion by host immune cells (80, 139). NDV can kill cancer cells through lytic viral infection as well (140). In the orthotopic, syngeneic murine GL261 glioma model, survival of treated animals was significantly prolonged with 50% long-term survival *versus* none in the control group. Immunogenic cell death was induced in GL261 cells after NDV infection (58).

Recombinant NDV (rNDV−p53) constructed of p53 oncolytic agent for the treatment of glioma improved the prognosis of mice with glioma due to inhibition of glioma cell growth and aggressiveness both *in vitro* and *in vivo* compared with rNDV or p53 alone. In addition, rNDV−p53 could induce apoptosis of glioma cells by upregulating apoptosis−related genes, stimulating lymphocyte infiltration and cytotoxic T lymphocyte (CTL) responses and increasing the number of apoptotic bodies *in vivo* (141).

In a Phase I/II trial, intravenous delivery of the oncolytic HUJ strain of NDV (NDV-HUJ) was well tolerated in patients with recurrent glioblastoma and one patient achieved a complete response (142). Currently, there are no active clinical trials using NDV as an oncolytic agent to treat GBM.

#### Vesicular Stomatitis Virus

VSV is a single-stranded, negative-sense RNA virus of the *Rhabdoviridae* family (81). Mild or asymptomatic infection of humans, rare and low pre-existing immunity to the virus and selective replication within cancer cells render VSV promising in OVT (143, 144). The strong neurotoxicity of VSV glycoprotein is however a major concern. A preclinical study replaced the VSV glycoprotein with the Chikungunya polyprotein E3-E2-6K-E1 to form a chimeric virus (VSVΔG-CHIKV), which appeared safe within the CNS. Furthermore, this chimeric virus could selectively infect brain tumors and prolong survival substantially in tumor-bearing mice through intracranial injection (145). Another variant, Vesicular Stomatitis Virus (VSV-ΔM51), could infect and kill both the TMZ resistant human brain tumor stem cells (BTSCs) and the differentiated compartments of GBMs *in vitro* (111). Recently, a novel recombinant VSV, G protein less (GLESS)-fusion-associated small transmembrane (FAST)-VSV, demonstrated antitumor effects in animal glioma models, providing the basis for clinical trials in the future (146).

#### Retrovirus

Retroviruses, belonging to *Retroviridae* family, are 100 nm in diameter containing two identical single-stranded, positive-sense RNA molecules 7–10 kilobases in length (82). TOCA 511 is an Moloney murine leukemia virus (MLV), encoding the cytosine deaminase (CD) gene to improve direct tumor cell killing *via* local conversion of prodrug 5-fluorocytosine (5-FC) to the active 5-fluorouracil (5-FU).

In a pre-clinical study, TOCA 511 was administered intravenously or intracranially in combination with 5-FC to treat immune-competent mice bearing glioma. Long-term survival and tolerability were observed, especially in animals with preexisting immunity to the virus, suggesting the potential for repeated administration (147).

Three Phase I clinical trial utilized TOCA 511 to treat recurrent high-grade gliomas (rHGGs) were completed (NCT01156584) (NCT01470794) (NCT01985256). In contrast to the promising results of the preclinical studies and the multiyear durable responses found in rHGGs patients in phase I clinical trials, no benefit was shown in a phase III randomized clinical study (NCT02414165) (148–150). One probable reason is that TOCA 511 is a recombinant oncolytic MLV, which can only spread efficiently in rapidly proliferating tumor cells in transplantable mouse tumors due to the short half-life of the virus in the cytoplasmic compartment, while slow proliferation is a hallmark of human cancers (151).

In a recent study, a replication competent Foamy Virus (oFV) was constructed with the ability to infect and replicate in slowly dividing tumor cells (151). This virus showed broad cancer tropism *in vitro*, including glioblastoma (U251).

#### Zika Virus

Zika virus (ZIKV) is a single-stranded, positive-sense RNA virus of the *Flaviviridae* family (83, 152, 153), most prominent for an outbreak in South and Central America that raised public health concerns globally, due to its potential to cause microcephaly in children of mothers infected during pregnancy (154). Irrespective of this concerning fact, ZIKV has oncolytic potential as it can cross the endothelial barrier and preferentially target glioblastoma stem cells (GSCs) (155, 156).

Preclinical findings indicate that Brazilian Zika virus strain (ZIKVBR) can selectively kill human malignant brain tumor cells (157). As it can selectively kill GSCs within the tumor, a live attenuated ZIKV vaccine candidate substantially reduced brain tumor growth and prolonged survival *in vivo*. Antiviral immunity, inflammation, and apoptosis of GSCs are stimulated through virus infection (158). A recent *in vivo* study showed that CD8+ T cells were required for ZIKV oncolytic activity and immune checkpoint inhibitors could improve the effect of OVT (159). Cytosine phosphate–guanine (CpG) recoding of Zika viral genome can reduce virus infection kinetics in nonmalignant brain cells, but retains high infectivity and oncolysis in GSCs (160).

A study using 3 dogs bearing spontaneous CNS tumors to evaluate the safety and therapeutic effect of Brazilian Zika virus (ZIKV^BR^) through intrathecal injection showed shrinkage of tumor, extension of survival and improvement of clinical symptoms without negative side effect (161). Further preclinical development of ZIKA OVT is required, including determinants of ZIKA infection in tumor cells, IFN signaling as well as protein expression signatures that enable viral entry and replication (162).

#### M1 Virus

M1 is a positive single-strand RNA virus, belongs to Getah-like alphavirus, *Togaviridae* family and was isolated from Hainan province in China (85). A number of studies found that M1 has a natural tropism to tumors which can be used for oncolytic virotherapy (163–165). It has significant anticancer activity including colon, bladder and liver cancer (164). M1 is safe and nonpathogenic for nonhuman primates after multiple rounds of repeated intravenous injections (62). More importantly, M1 has the ability to infiltrate the BBB, specifically suppress malignant glioma and prolong the survival time of glioma-bearing immunocompetent mice (166).

Infection with M1 induces the unfolded protein response (UPR) and subsequent autophagy. This UPR-autophagy axis can be blocked to significantly enhance the antitumor efficacy of M1 *in vitro* and *in vivo*. Expression of IRE1, a key element in the UPR pathway, is down regulated in higher-grade gliomas, suggesting favorable antitumor activity of M1in gliomas (167). No clinical trials with M1 in glioma have been conducted to-date.

#### Semliki Forest Virus

Semliki Forest virus (SFV) is another single-strand, positive-sense RNA virus belonging to alphavirus genus, *Togaviridae* family. SFV VA7-EGFP, an avirulent SFV A7 (92) strain with replication-competent capacity, has been evaluated *in vitro* in three human glioma cell lines (U172, U251, U87) and *in vivo* in subcutaneous and orthotopic tumor models in BALB/c mice. The three glioma cell lines were effectively killed and intravenously administered SFV VA7-EGFP completely eradicated 100% of small and 50% of large subcutaneously implanted U87Fluc tumors. Moreover, long-term survival was observed in 16 of 17 animals (86, 168).

miRNA expression is cell-type restricted which can be utilized for OVT to prevent replication of OVs in healthy tissues (169). To reduce neurovirulence and to target replicating tumor cells, tissue-specific micro-RNAs (miRNAs) were incorporated into multiple alphavirus vectors (169–171). Recently, an IFN-I tolerant SFV was constructed and tested in combination with ICIs in GL261 glioma model. Increased tumor-reactive CD8+ T cell infiltration in tumor microenvironment has been observed (172). No clinical trials with SFV in glioma have been conducted to-date.

#### Seneca Valley Virus

Seneca Valley virus isolate 001 (SVV-001) is a single-stranded, positive-sense RNA virus belonging to *Picornaviridae* family. It is nonpathogenic and can selectively infect and kill tumor cells, especially tumors with neuroendocrine features (87). As for brain tumors, α2,3- and α2,6-linked sialic acids were identified as necessary for SVV-001 infection in pediatric GBM cell lines. In an immunodeficient (SCID) mouse model of pediatric GBM, intravenous injection of SVV-001 significantly prolonged survival and completely eliminated xenograft tumors without infecting any normal cells in the brain (173). No clinical trials with SVV in glioma have been conducted to-date.

## Obstacles Faced by Oncolytic Virus Treatment of Gliomas

Although oncolytic virus therapy for gliomas is promising and many clinical trials are under way, there are still fundamental difficulties that need to be overcome:

1. Modulating OVs-mediated host immune response
2. Discerning radiographic progression from immunotherapy-induced pseudoprogression using neuroimaging;
3. Finding appropriate markers for therapeutic efficacy;
4. Identifying a suitable animal model;
5. Overcoming existing obstacles in OVs delivery

### Modulating OVs-Mediated Host Immune Responses

Pre-existing immunity was considered to be a hurdle for OVT as antibodies in peripheral blood would neutralize the virus particles and prevent OVs from reaching the tumor (174, 175). However, recent studies have shown that pre-existing immunity can actually boost the immune-mediated antitumor response, e.g., in NDV-immunized mice, anti-viral immunity to NDV improved tumor clearance, abscopal effects and mice survival, although virus replication within the tumor was limited (176). Even pre-existing immunity to other pathogens (tetanus) could be exploited to enhance the anticancer immune response triggered by oncolytic adenoviruses (177).

As for OVT induced immunity, finding a balance between the anti-tumor and anti-viral immunity remains a big challenge for viruses use as therapeutic agents. Immunosuppression can increase the distribution of the virus in the tumor, but it limits the ability of the immune system to kill the tumor. Strengthening the host’s immune system can enhance targeting of infected tumor cells, but it also limits the virus` distribution in the tumor site (178). An alternative strategy to prevent the injected OVs from clearance before they reach the target tissue are cell-based delivery platforms. These delivery platforms can provide shelter from the host immune response without suppression of the anti-tumor immune-response (94, 179, 180).

Attenuation is a viable strategy to avoid the toxicity of OVs and hyper activation of the host´s immune system. For example, the problem of high virulence and strong immunogenicity of ZIKV has been solved by creating a genetically modified attenuated vaccine variant of the virus that has low virulence and reduced immunogenicity but retains viral oncolytic activity against GBM (158).

Innate antiviral immunity is not just a hurdle for the efficacy of oncolytic viruses: type I IFNs can also play an important role in antitumor host immunity (181). In glioma, loss of type I IFN signaling promotes tumorigenesis (182). However, it is challenging to exploit enhanced type I IFN response to OV for OVT, but a number of approaches are being studied. One approach is to combine ICIs with OVT, as OVs can induce IFN release in the tumor microenvironment with upregulation of PD-L1 expression on tumor cells (70). Also, the local administration of a Semliki Forest virus encoding IL­12 (SFV-IL12) induces tumor-specific CTLs only when the host expresses IFNα/β receptor subunit 1(IFNAR1) (183, 184).

A recent study has found that stimulator of interferon genes (STING) pathway for DNA sensing has a major role in activating the adaptive immunity (by triggering type I IFN signals) against tumors, including gliomas (181, 185). Cytoplasmic DNA binding with cyclic GMP–AMP (cGAMP) synthase (cGAS), which in conjunction with STING, initiates the synthesis of type I IFN by immune cells (186). Batf3-lineage DCs respond to type I IFN, which can facilitate cross-presentation of antigens to CD8+ T cells (187, 188). In other words, STING is an important bridge between innate antiviral and adaptive antitumor immunity. It is reasonable to hypothesize that a curative therapeutic OV would be a virus that potently activates the innate immune system to trigger the antitumor adaptive immunity and resists the following antiviral response of the host (61).

There is a growing concern that OVT may be a double-edged sword with regards to tumor immunity. OVs can activate the immune system to fight the tumor through various mechanisms such as antigen-presentation, cytokine release and gene delivery. However, a growing body of evidence suggests that these mechanisms, while activating immunity, also have immunosuppressive effects. For instance, GM-CSF recruits macrophages, but whether these macrophages are present in the form of M1 or M2 remains in question (189). Also, OVs can upregulate PD-L1 expression on tumor cells *via* the induction of IFN release in the tumor microenvironment, enabling tumor escape from the immune system (15). Furthermore, some OVs like the vaccinia virus and VSV possess natural immune escape mechanisms, which also have dual effects on cancer treatment (190, 191). These mechanisms can prevent virus particles from neutralization, increasing replication and treatment efficacy on one hand, but at the same time they may not be able to adequately activate anti-tumor immunity on the other. Simply put, OVs activate the immune system, but they also suppress it to establish themselves. This might be the main reason that the efficacy of OVTs as monotherapies, including T-Vec, is not very satisfactory. Each OV influences a unique, heterogeneous immune microenvironment. To improve OVT further, we should find the appropriate adjuvant therapy for each oncolytic virus and enhance the therapeutic effect based on the underlying mechanisms.

### Discerning Radiographic Progression From Immunotherapy-Induced Pseudoprogression Using Neuroimaging

Pseudoprogression is a big challenge in the follow-up of glioma patients that remains unsolved at present. It can be detected in 10–30% of GBM patients on their first MRI, which shows oedema and sometimes contrast enhancement after undergoing radiotherapy and concurrent TMZ in the first 12 weeks (192–194). The target lesions in these patients continue to grow on their first MRI and then become stable, shrink, or even disappear during the subsequent imaging follow-ups. In addition, pseudoprogression has also been observed in cancer patients undergoing immunotherapies (e.g. ICIs and OVT) due to the increased immune cell infiltration (195–197).

Pseudoprogression challenges the interpretation of results and decision-making in clinical trials (198). Steroids are commonly used to control oedema and increased intracranial pressure during the stage of pseudoprogression, but long-term use can cause substantial side-effects (199). Furthermore, steroids are likely negatively affecting any immunotherapeutic interventions due to their well-known immunosuppressive effects (200). The pathophysiology of pseudoprogression and the associated molecular changes have not been fully understood yet and require further studies.

At present, RECIST 1.1 is the gold standard for assessing treatment response in solid tumors including gliomas but has not fully succeeded in overcoming the challenge of pseudoprogression during immunotherapy (201). A consensus guideline, iRECIST, developed by the RECIST working group tries to address this problem (202). In addition to iRECIST, Response Assessment in Neuro-Oncology (RANO) criteria also takes pseudoprogression in gliomas into consideration (203). With the development of radiologist´s expertise and further experimental tweaks, researchers are expected to overcome the problem of pseudoprogression soon (204–206).

### Finding Suitable Markers for Evaluating the Effectiveness of Therapy

Currently, no validated biomarkers of OVT exist. However, some biomarkers like the components of antiviral and antitumor immune response pathways, including IFN signaling elements, cGAS–STING, retinoic acid-inducible gene I (RIG-I), melanoma differentiation-associated gene 5 (MDA5), and various Toll-like receptors (TLRs) are showing great promise in OVT (207). In a recent study, impaired immunogenicity was shown in STING-knockout cancer cells after oncolytic HSV-1 infection. In immunocompetent models, STING-knockout tumors were more resistant to treatment with oncolytic HSV-1 combined with PD-1 blockade. With a partial or complete loss-of-function STING genotype, patients may not take full advantage of OVT, at least for HSV-1 (185). Therefore, STING could potentially be used as a biomarker for the screening of patients to identify those who can benefit from OVT. A logical next step is to analyze the expression of genes involved in the STING pathway in glioma patients and the effect of STING pathway in other OVTs.

In addition to cGAS/STING pathway for foreign DNA sensing, cells equipped with RIG-I/MDA5 pathway, which mainly serve for foreign RNA sensing (208). Both RIG-I and MDA5 have been observed to trigger a robust innate immune response against various tumors and effectively counteracts tumor cell heterogeneity, especially in human primary GBM (209–212). TLRs are the most widely studied pattern-recognition receptors. Targeting TLRs can have anti-tumor activity by promoting antigen presentation and activating innate and adaptive immunity (213, 214). All three, RIG-I, MDA5 and TLRs can be activated by some OVs, providing the possibility for combining agonist of these receptors with OVs (215–219). However, the combination therapy has to avoid sensitizing the patient to a cytokine shock-like response induced by IV delivery of OVs (220). Also, some receptors like TLRs are expressed on glioma cells with tumor-promoting properties, which should be taken into account when developing agonists into cancer immunotherapeutic (221).

Several other potential biomarkers are under investigation, including the number, density and localization of immune cells (NK cells, DCs, T_reg_ cells, CD4^+^/CD8^+^ T cells), the levels of checkpoint molecules (PD-L1, CTLA-4, LAG-3), the presence of viral antigens in tumors and other pathways like IRE1 (15, 74, 134, 167, 222, 223).

Currently, since OVT biomarkers in glioma have not been validated in clinical trials, they usually do not affect trial enrollment. OVs are not usually based on specific markers because they are not just targeting one molecule during the infection. The complex mechanism of OV makes the prospective use of a single marker insufficient to assess the therapy success.

Using OVs with knowledge of the specific mechanisms of their action will greatly benefit the OVT: appropriate markers can provide information about whether these mechanisms are working or not. More details on potential biomarkers for OVs in GBM were recently reviewed by Stavrakaki et al. (224).

### Identifying a Suitable Animal Model

Clinical trials are guided by preclinical studies. Therefore, it is very important to apply the models that are most suitable for clinical research. An ideal experimental model of glioma should meet the following requirements: 1) tumor microenvironment that resembles human tumors in the brain; 2) genetic background that is similar to human gliomas; 3) a well-developed immune system that resembles human immunity; 4) intratumoral heterogeneity; 5) manipulability; 6) reproducibility; 7) cost-effectiveness; 8) ethical compliance (225). Currently, the majority of the preclinical studies for glioma include syngeneic models, xenografts models, genetically engineered models (GEMs) and resection models. Although each has different advantages and disadvantages, none of them can meet all of the above requirements (226–231).

It is worth mentioning that established glioma cell lines and primary glioma tumors (highly heterogenous) have substantial differences in both genomic alterations and gene expression, indicating that glioma cell lines may not be an ideal model system for primary gliomas (232). Until now, rodents have been commonly used for most preclinical studies, however, they are not the best models for assessing OVT. Some OVs are species-specific and unable to efficiently infect and replicate in murine cells (e.g. human specific adenoviruses) (233). Consequently, the use of xenografts may offer a solution to this problem, but the complexity of the tumor microenvironment is not reproduced. A study using Syrian hamster as an immune-competent model found that this model could support the replication of both human-adenovirus and vaccinia virus.

New glioma models are emerging that provide more opportunities for preclinical research. These models include new ways of organizing cells outside the body, such as organoids, that better mimic natural gliomas. They also include new organisms such as zebrafish, fruit flies and dogs (234–237).

### Overcoming Existing Obstacles in OVs Delivery

Current delivery approaches include local delivery (intracavitary, intrathecal, and intratumoral delivery) and systemic delivery (intravascular delivery) (238).

**Figure 3:**
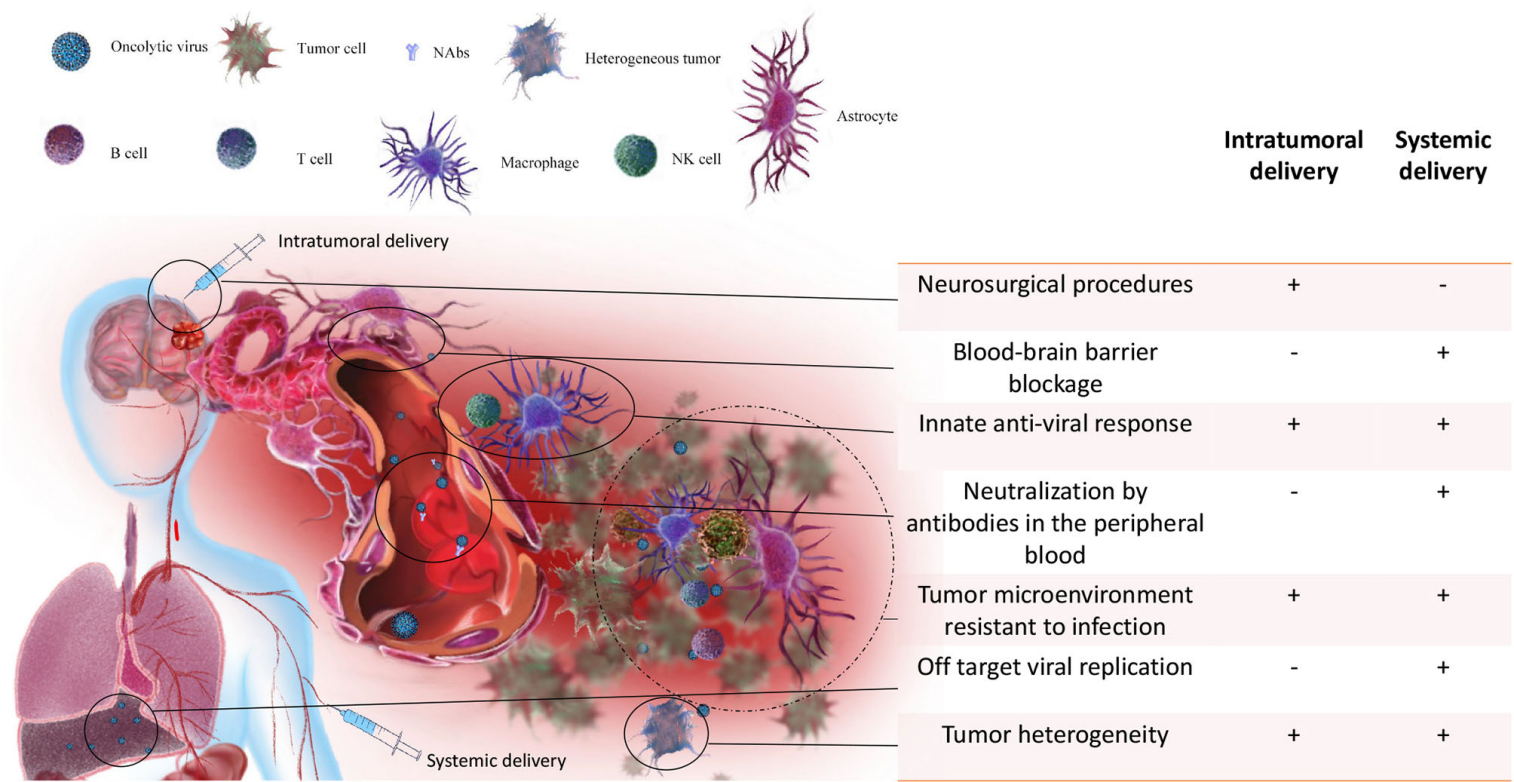
Obstacles to OVT of gliomas *via* intratumoral and systemic delivery. For intratumoral delivery only, 1) the expense and complexity of neurosurgical procedures, limiting repeat administration. For systemic delivery only, 1) After injection, the first challenge is neutralization of OVs by antibodies in the peripheral blood; 2) Off target effect is the second challenge for systemic delivery since OVs may not be able to reach to the brain and cause infection to normal tissue (e.g. liver); 3) even if OVs can reach to the brain, the intact BBB is able to block the passage of most viruses. For both intratumoral and systemic delivery, 1) Innate anti-viral response can prevent OVs from interacting with tumor cells; 2) the specific tumor microenvironment is also resistant to OVs infection and suppresses the OVs-induced anti-tumor immune response; 3) tumor heterogeneity can make OVs insensitive to part of tumor cells.

Many OVs have been studied in patients with glioma by intracavitary or intratumoral injection, including HSV-G207, HSV-1716, adenovirus-dl1520 (ONYX-015) and reovirus. Intracavitary delivery is an established way to treat gliomas, but it has been limited by the poor penetration ability of drugs reaching only tumor cells adjacent to the surgical cavity. Therefore, intracavitary delivery usually requires a maximal surgical resection (239). Intratumoral delivery is the primary way for most oncolytic viruses in clinical trials of glioma treatment, including adenovirus, herpes simplex virus, measle virus and enterovirus (240). Studies show that intratumoral injection can not only induce tumor lysis in the injected region but also trigger systemic antitumor immunity of the whole body (59, 123). Direct injection of oncolytic viruses into tumors primes T cells specific to virus components as well as tumor cells and induces local inflammation. This inflammatory condition allows recruitment of more T cells, thus promoting recognition and destruction of tumors (241). Intratumoral delivery requires careful patient selection, and technically challenging neurosurgery, limiting repeat administration (**Figure 3**) (15). Another challenge is to balance the degree of local immunosuppression which is discussed in section “*Modulating OVs-Mediated Host Immune Responses*” CED is an intratumoral delivery using continuous, low–positive-pressure bulk flow to deliver drugs through the implantation of catheters. The difficulty of delivering repeated doses and the uncertainty in achieving meaningful drug concentrations throughout the brain makes this approach challenging (92). In the future, OVT may be based on local, image-guided delivery, which will allow direct visualization of the injection site, maximizing the availability and potential effectiveness of the OVs (242).

Systemic vascular delivery is becoming an option for more and more OVs. Current studies have found that several viruses, including reovirus, vaccinia virus, Newcastle disease virus, parvovirus H-1, chimeric vesicular stomatitis virus, and M1 virus are able to infect brain tumors in animal models after systemic vascular delivery (61). In addition, clinical studies demonstrated that parvovirus H-1 and reovirus can reach the brain after systemic delivery (15, 60, 110). Compared with local delivery of OVs, systemic delivery can better stimulate immune responses throughout the body, particularly in metastasis. Because of the known heterogeneity of tumor cells, it is important to stimulate antitumor responses to a wide range of tumor associated antigens (TAAs) (243). However, there are many difficulties associated with systemic delivery that have yet to be overcome (**Figure 3**):

1. The intact BBB is able to block the passage of most viruses;
2. Even if the BBB is disrupted, the host immune system outside the CNS can neutralize the viruses;
3. Off target effects may lead to the infection of host tissue.

Pre-clinical studies have demonstrated that low-intensity pulsed ultrasound in combination with systemically injected microbubbles could temporarily open the BBB in a localized manner (244). This concept is currently undergoing clinical trials, and has already demonstrated safety and tolerability in patients, and therefore may provide enhanced systemic OV delivery capabilities (245, 246).

It is worth paying attention to antiviral antibodies that already exist in patients, as they can be an obstacle to the systemic delivery of the virus. Cellular carriers can protect some OVs (eg, reovirus) from neutralizing antibodies (76). However, more and more studies show that preexisting immunity to OVs can enhance their therapeutic efficacy by inducing a more robust antitumor immune response, which has been observed not only with systemic delivery, but also with local delivery (176, 247).

Recently, a peptide-delivery platform for targeting malignant brain tumor in an immunocompetent syngeneic murine GBM model was developed. The approach enhanced targeted OV delivery and therapeutic efficacy (248). It is promising that with more clinical evidence in the future, negating the requirement for repeated invasive cranial procedures, systemic delivery may provide safer and more effective access to gliomas.

There is a shared problem of both local and systemic deliveries: the heterogeneous genotype/phenotype makes the tumor microenvironment challenging for viral replication (249). Diverse levels of IFN response in tumor microenvironments due to tumor heterogeneity make tumor cells in which the antiviral pathway is functioning well, insensitive to OV (250). The combination of several oncolytic viruses, as well as the combination of OV and other immunotherapies based on a different anti-tumor mechanism, may be a viable approach to address the problem of tumor heterogeneity. Overall, multiple approaches are currently undergoing clinical trials to overcome significant difficulties in efficient OV delivery to the CNS.

## Combination With Other Immunotherapies

Immunotherapy is a well-established cancer therapy. The following types of immune therapies are used in clinics for treating brain tumors: immune checkpoint inhibitors, anti-tumor vaccines, adaptive T-cell transfer and cytokines (251). Gliomas have immunosuppressive microenvironments, that limits the effectiveness of traditional immunotherapy. The infection and replication of oncolytic viruses in tumors can activate the anti-tumor immunity and turn “cold” tumors into “hot” ones (252). More and more evidence suggest that oncolytic viruses can enhance the effect of immunotherapy. In preclinical studies, many OVs launched an effective antitumor response against TMZ resistant glioma (111, 117). Also, non-overlapping mechanisms have been found between OVs and other immunotherapies (253). These findings suggest that there may be no cross-resistance to OVs and other glioma treatment.

### Immune Checkpoint Inhibitors

Immune checkpoint inhibitors (ICIs) are monoclonal antibodies that inhibit cytotoxic T lymphocyte antigen 4 (CTLA4) or PD1 and its ligand PDL1 to reactivate the immune system against tumor (254). Several phase III clinical trials of ICIs for the treatment of glioblastoma are underway, including therapy with ipilimumab to block CTLA-4 and nivolumab to block PD-1 (NCT02017717, NCT02617589). However, in the treatment of patients with recurrent GBM, no significant differences in overall survival (OS) were shown when comparing nivolumab (anti-PD1) and bevacizumab (anti-VEGF) (255). Brain metastases respond better to ICI compared to primary brain tumors (NCT02320058). Probable reasons for the limited response to ICIs are: relatively low mutant load, little T cell infiltration and immunosuppressive microenvironment in GBM (10). In addition, infiltrating PD-1-expressing T cells are rare in GBM, and tumor cells rarely express PD-L1, making for a “cold” tumor microenvironment (9).

OVs can enhance the immune responses by increasing infiltration of immune cells to tumors and improve the efficacy of ICIs, thus reconditioning the tumor microenvironment and transforming a “cold” tumor microenvironment into a “hot” one (52, 252). Studies suggest that OVs can be used to prime the immune response initially to create a ‘hot’ tumor microenvironment and make brain tumors sensitive to subsequent ICIs treatment (15, 256). Moreover, OVs can upregulate PD-L1 expression on tumor cells *via* the induction of IFN release into the tumor microenvironment, thus improving the therapeutic response to ICIs (15). Also, OVs can be engineered to express PD-L1 inhibitors, which can activate tumor neoantigen-specific T cell responses (257). In an immunocompetent mouse model of glioma, OVs combined with ICI have shown promising results. Among these viruses in combination with anti-PD-1 were tested adenovirus Delta-24-RGDOX, HSV (G47D-mIL12), VSV, reovirus, and measles virus (134, 258–260).

Of note, a multi-institution randomized clinical trial using neoadjuvant anti-PD-1 immunotherapy for the treatment of recurrent glioblastoma has shown positive results. The trial was conducted by The Ivy Foundation Early Phase Clinical Trials Consortium. OS was significantly longer for neoadjuvant plus adjuvant pembrolizumab compared to adjuvant pembrolizumab alone. Interferon- and T cell-related gene expression was upregulated while cell cycle-related gene expression was downregulated within the tumor only in the neoadjuvant group (261). All these findings suggest that scheduling of ICI and OV therapy might be of major advantage and should be explored in future trials. Overall, ICIs are the most promising therapy for combination with oncolytic viruses. Since ICIs can affect OV-induced antiviral and antitumor immunity, further studies of the immune responses triggered by these agents could significantly contribute to the development of OV plus ICI combination therapy (61).

### Cancer Vaccines

There are several types of cancer vaccines, including cell-based, protein, peptide, and genetic vaccines (249). Cancer vaccines against brain tumors have broad application potential. However, most tumor vaccines fail to recruit sufficient T helper cells and display reduced MHC II epitope on DC surfaces, thus lacking support to the enhancement of antitumor T-cell immunity (262, 263). The search for an optimal strategy for the use of antitumor vaccines to enhance antitumor T-cell immunity is the main direction of research in this area. The combination of vaccines with oncolytic viruses is a promising therapeutic approach, since T-cell immunity can be further improved with virotherapy (264).

#### Dendritic Cell Vaccines

In phase I and II trials, DC vaccines increased cytokine production in patients with gliomas after treatment and improved survival (265). The most advanced DC vaccine currently in clinical trials is ICT-107, which has been shown to be safe and effective. In a phase II study of 124 patients with brain tumors, compared with a control group, ICT-107 significantly improved progression-free survival (PFS) while maintaining QoL (quality of life) (266, 267). To maximize the immunogenicity of DCs, the focus is on optimizing the culturing conditions for DC generation *in vitro* (268). Additionally, preclinical studies have shown that oncolytic HSV-1 in combination with immature myeloid dendritic cells (iDCs) can reduce tumor volume and prolong survival by enhancing antitumor immunity in murine malignancies. OVs have the potential to enhance the therapeutic effects of cancer vaccines in combination therapy (269–273).

#### Peptide Vaccines

A specific tumor antigen that can be targeted by peptide vaccines can be represented by the following genes EGFRvIII, IDHR132H, Wilms tumor 1 (WT1) and survivin. In a phase II trials, a peptide vaccine targeting EGFRvIII improved survival in glioblastoma substantially, but the effect could not be replicated in a randomized phase III trial (8, 274). In a non-randomized trial, another peptide vaccine against WT1 improved the survival of patients with glioblastoma by stimulating the anti- WT1 IgG responses (275). Further, in a preclinical study, synthetic long peptides (SLPs) combined with oncolytic Maraba virus (MG1-E6E7) showed a significant anti-tumor effect against advanced HPV positive neoplasia. MG1-E6E7 expresses a tetravalent transgene, which is based on attenuated viral oncogenes E6 and E7 from HPV16 and 18. HPV-associated cancers may become an attractive target for peptide vaccination through the expression of these transforming viral oncogenes. MG1-E6E7 substantially enhanced the specific CD8+ immune responses induced by SLP vaccination (276). It is expected that the combination with OV will soon be tested on gliomas. However, antigenic loss is a substantial obstacle to single-peptide vaccinations. For example, several studies have shown that most patients gradually lost EGFRvIII expression after treatment, leading to tumor recurrence (274). Therefore, multi-peptide vaccines may be required to target glioma variable antigens to improve treatment efficacy (277, 278).

Recently, phase I clinical trials of glioblastoma have shown that neoantigens vaccines can induce intratumoral T-cell responses (279). This could represent an interesting alternative approach to improve the effectiveness of OVT treatment in glioma.

### Adoptive Cell Therapy

ACT holds considerable promise for the treatment of brain tumors. T cells are extracted from patients, cultured and proliferated *in vitro* to improve their ability to recognize and kill tumor cells, and then injected into patients. ACT includes tumor-infiltrating lymphocytes (TILs) and genetically engineered T cells. TILs have been reported to induce regressions in some tumor types, but it is difficult to isolate and expand TILs from the CNS (18). The administration of autologous CMV- specific T cells offer a new way to treat brain tumors (280). However, chimeric antigen receptor (CAR) T cells therapy for brain tumors still faces significant challenges, such as heterogeneity of target antigens in tumor cells, induction of compensatory immunosuppressive response in the brain and failure to recruit infused T cells into brain tumors (281–284). Better strategies are needed to assist T cells in efficient infiltration, overcoming the immunosuppressive GBM microenvironment and handling the immune-related complications associated with ACT (10).

Combination with emerging OVs offers massive opportunities for ACT to overcome these obstacles. OVs can assist in the recruitment and activation of selected or engineered T cells in the tumor bed (285). Also, OVs can up-regulate the expression of MHC class I molecules by tumor cells, which can improve the targeting of ACT to tumor-specific antigens (286). In murine neuroblastoma models, arming adenovirus with the chemokine like CCL5/RANTES and the cytokine IL-15 not only preserved their oncolytic effects but also enhanced the migration and proliferation of the tumor- associated ganglioside GD2 CAR T cells, thereby increasing overall survival (287, 288).

Unexpectedly, another pre-clinical study demonstrated that OV-associated type I IFN response has negative effect on CAR T cell therapy (289). The inflammatory environment generated by the oncolytic viruses and the remodeling of the tumor microenvironment neither helped in recruiting CAR T cells nor enhanced their functionality. The concentration of type I IFN in the tumor was inversely correlated with the number of the CAR T cells. Furthermore, IFNAR1 knock out CAR T cells showed resistance to the negative effects caused by OVs in the setting of lymphodepletion or NK cell depletion. A solution may be to limit the type I IFN signaling associated with an OV or making CAR T cells insensitive to type I IFN. Serving as soluble type I IFN decoy receptor, the expression of B18R gene in vaccinia virus may improve the combination of oncolytic vaccinia virus with CAR T cells in a TC1-mesothelin model (289, 290). Negative effects on ACT efficacy are not the only effects of type 1 IFN that can be caused by OV in tumor. There may be other effects as well. The key role of type I IFN in tumors should be considered more broadly with OV re-dosing when the endogenous T cell compartment may be depleted. For the time being, there are few experimental studies on the combination of ACT with oncolytic viruses in gliomas. However, these studies show that the combined approaches of OV and CAR T cells are promising for the treatment of brain tumors.

### Immunotherapeutic Modulators

Multiple OVs have been armed with different anti-tumor cytokines to activate the tumor microenvironment and enhance anti-tumor immunity induced by OVs (98).

IL-12 can produce multifaceted anti-tumor effects but has been shown to have serious adverse effects when administered intravenously (291, 292). Recent studies found that OVs can limit the systemic toxicity of IL-12 by expressing IL-12 locally in the tumors as well as in the brains of non-human primates (293, 294). In a murine GSC model, oncolytic HSV-1 encoding IL-12 (HSV-1-IL-12) replicated in GSCs *in vitro* and enhanced survival of syngeneic mice bearing GSC-derived tumors *in vivo*. Furthermore, HSV-1-IL-12 can activate anti-tumor immune response through increasing IFN-γ release and reducing the number of regulatory T cells in the tumor (98). At present, in a phase I clinical trial, an engineered HSV-1 expressing IL-12, to treat recurrent glioma is recruiting volunteers (NCT02062827).

The gene for granulocyte macrophage colony stimulating factor (GM-CSF) has been used in several OV constructs. GM-CSF enhances the activation of NK cells and CD8-mediated T cell response downstream in OVT by promoting maturation of monocytes in DCs and improving the DC’s ability to present antigens (295, 296). Many other constructs are also being investigated, including HSV-1-IL-4, VACV-CCL5, VSV-IFNg and VSV-IL-15, ADV-IL-15 (259, 270, 297–301). There is no doubt that OVs encoding immunotherapeutic modulators for the treatment of malignant gliomas will become more and more popular in clinical studies.

## Future Directions

Because of the complex interactions between the tumor and its host, gliomas remain lethal and conventional therapies have improved little in recent years. Gliomas are heterogeneous tumors and tend to be difficult to target for immunotherapy since they contain few mutations (18). Immunotherapy can only be successful if its effect reaches the brain and overcomes the difficulties associated with tumor heterogeneity and tumor targeting. The most significant result of OVT clinical trials is that this therapy is well tolerated by patients with glioma and has relatively rare serious side effects. Overall, OVT offers a selective, innovative and safe approach to treating glioma (44). Furthermore, antigen-specific T cells in gliomas display an exhausted phenotype (302, 303). OVs have the potential to change the phenotype of T cells from exhausted to activated type and provide more possibilities for the combination of an oncolytic virus with other immunotherapies (59). However, the mechanisms of interaction between tumor, host and OVs in brain tumor microenvironments have not been fully elucidated. Further study is needed to maximize the efficacy of OVs.

As discussed in section “*Modulating OVs-Mediated Host Immune Responses*”, OVT may be a double-edged sword because OVs can not only activate the immune system but also suppress it to establish themselves. Different viruses can create different tumor microenvironments. Therefore, in the future, improving OVT by finding the appropriate adjuvant therapy for each OV and enhancing the therapeutic effect considering the underlying mechanisms should be the norm. In principle, two ways can be used to achieve this: combination therapy and arming the OV with adjuvant factors.

At the same time, personalized therapeutic approaches are becoming feasible for OVT. An ideal combination of appropriate viruses could be chosen according to the type, stage and biomarker expression of glioma. The success of oncolytic virotherapy may not only require careful screening of patients based on different mutations and protein expression in their tumors, but also require the selection of suitable OVs for a particular tumor. An *ex vivo* 3D tumor model generated from fresh tissue provides a culture condition that supports further researches into the dynamics of viral infection and the interaction with local immune system in the specific tumor microenvironment (304). With application of this system, it is possible to screen multiple OVs for a specific patient and establish the optimal OVT (224). Undoubtedly, identifying the optimal dosing, route of administration and schedule in OVs treatment requires further investigation. The sequence of application of different agents involving OVs is still under investigation. Well-designed clinical trials will pave the way for suitable, effective, and precise OV glioma treatments in the future.

Approaches are being developed that combine oncolytic virotherapy and many other types of immunotherapies. As two or more agents are combined, there is always a concern about the increased overall toxicity. Therefore, it is important to find more synergistic combination therapy modalities and to further understand the mechanisms of each therapy and clinical effects on immune response (305). Currently, more and more combinatorial clinical studies are under way, which undoubtedly indicates that the application of oncolytic viruses in glioma has great prospects (**Tables 2**, **3**).

## Conclusions

The development of OV for the treatment of gliomas has been going on for over two decades, and the potential of this therapy is being recognized in an increasing number of studies. As a special immunotherapy, OVT can not only kill gliomas directly, but can also activate the body’s effective anti-tumor immunity, which can cause a synergistic effect with other immunotherapeutic methods. However, much remains to be learned about OVT and combination therapy, including the mechanisms that mediate the immunosuppressive microenvironment of brain tumors, optimal OVs intratumoral delivery, selection of most appropriate OVs and immune targets for different tumors, and molecular markers for prediction of therapeutic efficacy. The approval of G47Δ (Delytact/Teserpaturev) for the treatment of glioma in Japan, although time-limited and conditional, is the culmination of decades of OVT research in glioma. Undoubtedly, oncolytic viro-immunotherapy of gliomas has great prospects and, in the future, will have a great impact on cancer therapy.

## Author Contributions

JZ: Conceptualization and writing and editing of the manuscript draft. XL: Drawing of the electronic illustrations. MS: Review and language editing of the manuscript. HZ: Review of the manuscript. GY: Instruction and review of the manuscript. YL: Instruction and review and editing of the manuscript draft. All authors contributed to the article and approved the submitted version.

## Funding

This research was funded by National Natural Science Foundation of China (grant number 81973347), Guangdong Basic and Applied Basic Research Foundation (grant number 2019A1515011564) and Pearl River S&T Nova Program of Guangzhou (grant number 201906010069).

## Conflict of Interest

Author MS was employed by company Guangzhou Virotech Pharmaceutical Co., Ltd.

The remaining authors declare that the research was conducted in the absence of any commercial or financial relationships that could be construed as a potential conflict of interest.

## Publisher’s Note

All claims expressed in this article are solely those of the authors and do not necessarily represent those of their affiliated organizations, or those of the publisher, the editors and the reviewers. Any product that may be evaluated in this article, or claim that may be made by its manufacturer, is not guaranteed or endorsed by the publisher.
